# A Submarine Journey: The Pyrrole-Imidazole Alkaloids †

**DOI:** 10.3390/md7040705

**Published:** 2009-11-27

**Authors:** Barbara Forte, Beatrice Malgesini, Claudia Piutti, Francesca Quartieri, Alessandra Scolaro, Gianluca Papeo

**Affiliations:** 1 Department of Medicinal Chemistry, Nerviano Medical Sciences, Viale Pasteur 10, 20014 Nerviano, Milano, Italy; E-Mails: barbara.forte@nervianoms.com (B.F.); claudia.piutti@nervianoms.com (C.P.); alessandra.scolaro@nervianoms.com (A.S.); 2 Department of Chemical Core Technologies, Nerviano Medical Sciences, Viale Pasteur 10, 20014 Nerviano, Milano, Italy; E-Mails: beatrice.malgesini@nervianoms.com (B.M.); francesca.quartieri@nervianoms.com (F.Q.)

**Keywords:** pyrrole-imidazole alkaloids, total synthesis, marine sponges, oroidin

## Abstract

In his most celebrated tale “The Picture of Dorian Gray”, Oscar Wilde stated that “those who go beneath the surface do so at their peril”. This sentence could be a prophetical warning for the practitioner who voluntarily challenges himself with trying to synthesize marine sponge-deriving pyrrole-imidazole alkaloids. This now nearly triple-digit membered community has been growing exponentially in the last 20 years, both in terms of new representatives and topological complexity – from simple, achiral oroidin to the breathtaking 12-ring stylissadines A and B, each possessing 16 stereocenters. While the biosynthesis and the role in the sponge economy of most of these alkaloids still lies in the realm of speculations, significant biological activities for some of them have clearly emerged. This review will account for the progress in achieving the total synthesis of the more biologically enticing members of this class of natural products.

## 1. Introduction

Sometime in 2003, our Company decided to set up a new project based upon the chance of fishing out new potent and selective kinase inhibitors from the natural products armoury. Particularly, the task was to play with the marine-origin metabolite hymenialdisine (see Subsection 3.1), a natural-born *pan*-kinase inhibitor. The goal of that project would have been to first synthesize this pyrrole-imidazole alkaloid and then to bias its biological activity through chemical manipulations. A non-biomimetic, simple disconnection allowed us to cut off the double bond connecting the northern glycocyamidine ring with the southern pyrroloazepinone, thus envisioning aldisine as the suitable key intermediate. However, as soon as we were able to routinely produce multigram batches of this intermediate, an outstanding paper from Wan *et al*. [1] (see Subsection 3.1) was published, in which a number of hymenialdisine derivatives, even larger than in our rosiest hopes, was synthesized. Despite this setback, we pursued anyway the opportunity to successfully complete the hymenialdisine synthesis. From then on, the temptation of challenging other pyrrole-imidazole alkaloids was hard to resist, as much as it was hard to resist the enjoyment of the stimulating literature dealing with those natural products, published during the last five years in exponentially growing numbers.

The idea of a review recounting the efforts and achievements in the total synthesis of pyrrole-imidazole alkaloids was actually born from the perusal of these papers. However, as a number of reviews dealing with this topic are already available [2–10], we decided to cover the literature from 2005 to mid-2009 by focusing only on those alkaloids possessing a clear pharmacological value. A brief discussion on the biogenesis and the ecological role of these natural products will be the *hors-d’œuvre*.

### 1.1. Pyrrole-Imidazole Alkaloids Sources and Biogenesis

**Figure 1.**
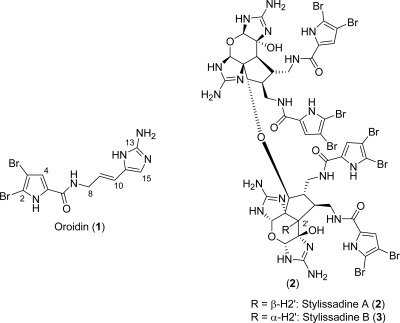
PIAs complexity extremes.

The pyrrole-imidazole alkaloids (PIAs) family comprises hundreds of secondary metabolites originating from marine sponges exclusively. Those natural products, whose architectural complexity goes from simple, achiral, monomeric oroidin (**1**, Figure 1) to the breath-taking 16-stereocenter-containing tetrameric stylissadine A and B (**2** and **3**, Figure 1), have been mainly isolated from various species of *Agelasidae*, *Axinellidae*, *Dyctionellidae* and *Hymeniacidonidae* families of sponges [2]. The systematic recurrence of PIAs in these families of sponges allowed to speculate their taxon-specificity and then to consider these secondary metabolites as chemical markers for phylogenetically related sponges [11,12]. The localization of those alkaloids in sponge cells has recently been investigated [13].

**Figure 2.**
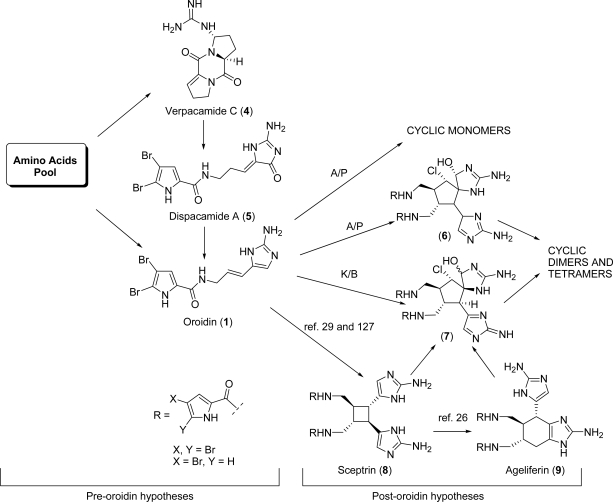
PIAs biogenetic speculations.

There are numerous speculations in the literature on the biogenesis of PIAs. As the simple oroidin (**1**, Figure 1) is mostly considered to be the biogenetic precursor of any other alkaloid pertaining to this family, those hypotheses can be grouped according to their pre- or post- oroidin focus. Pre-oroidin speculations are based on the fundamental aminoacids involved in the production of oroidin in living organisms (Figure 2). Thus, proline and/or ornithine may provide the backbone to build up the pyrrole-2-carboxylic acid moiety, while histidine may contribute to the 2-aminoimidazole portion. Low incorporation of these three aminoacids in the biosynthesis of the oroidin cyclized derivative stevensine has been experimentally observed by Kerr *et al*. by feeding cell cultures of the marine sponge *Axinella corrugata* (previously *Teichaxinella morchella*) with the corresponding ^14^C-labeled aminoacids [14]. Alternatively, ornithine [11,12] as well as lysine [15] may be the source of the the five contiguous carbon atoms present in the aminopropenylimidazole portion of oroidin. A more recent and intriguing hypothesis emerged upon the isolation of verpacamides A-D (e.g. verpacamide C, **4**, Figure 2) from the marine sponge *Axinella vaceleti* [16].

These cyclo(l-Arg-l-Pro) dipeptides differ only by their oxidative state, thus resembling intermediates along a given biogenetic pathway. The authors, by capitalizing on the discovery of an unprecedented conversion of proline into 2-aminoimidazolone [17], speculate that verpacamides may represent the biogenetic precursors of dispacamide A (**5**, Figure 2), a postulated forerunner of oroidin.

Post-oroidin hypotheses, on the other hand, can be sub-classified according to the alkaloids and their respective biosynthesis. Thus, along with a number of biochemical considerations on single natural products, frequently formulated while disclosing their isolation [18–28], more general speculations that try to link as many PIAs as possible have emerged. The seminal paper in this respect was published in 2001 by Al Mourabit and Potier (A/P) [29]. The ability of a given enzyme, through proton exchange reactions, to govern the tautomeric equilibria (and consequently the corresponding nucleophilic/electrophilic behaviour) of the aminopropenylimidazole portion of oroidin, was hypothesized to be the key point for the formation of both polycyclic monomers and cyclized dimers. The common intermediate (**6**, Figure 2) arising from oroidin that the authors invoked for the formation of the palau’amines, the styloguanidines and the axinellamines groups of alkaloids (see Section 5), was subsequently elaborated by Köck, Baran *et al.* (K/B) from both a stereochemical and an oxidative state point of view [30], in light of the recently revised structure of palau’amine (see Section 5). The symmetry that this revision finally brought to the cyclized dimeric members of the PIAs family, allowed the so-called “pre-axinellamines” intermediate (**7**, Figure 2) not only to more precisely connect the aforementioned groups of natural compounds, but also to include the konbu’acidins and the subsequently isolated massadines and stylissadines in this network. According to the same hypothesis, the “pre-axinellamines” **7** may be derived, in turn, from oroidin (**1**), sceptrin (**8**, Figure 2) or from ageliferin (**9**, Figure 2) [30]. Interestingly, the amide hydrolysis that Köck, Baran *et al*. invoked in order to cleave redundant bromopyrrole moieties in the styloguanidine and palau'amine biosynthetic hypothesis (see Section 5) [30], is in agreement with the supposition that the 4,5-dibromopyrrole-2-carboxylic acid derives from oroidin hydrolysis, and is not one of its precursors [17].

If, on the one hand, nothing is known about either the enzymes or the corresponding genes by which sponges perform the aforementioned biogenetic transformations, on the other hand, haloperoxidase-mediated introduction of halogen(s) onto those marine alkaloids are easy to be inferred. Those enzymes, whose cofactors are represented by heme iron or vanadium, employ hydrogen peroxide to generate a metal-bound hypohalite ion, that is the electrophilic halogen source responsible for the halogenation of electron-rich substrates like the pyrrole nucleus in PIAs [31,32]. Other, recently discovered, haloperoxidases (e.g., PrnA) use flavin adenine dinucleotide as a metal-free cofactor to halogenate nucleophilic substrates [33].

The absolute configuration of many of the more complex members of the PIAs family is still not known. Despite the fact that tentative assignment through CD spectroscopy (for a recent example, see Nishimura *et al.* [34]) for some of the simpler representatives has proven to be correct [35–37], only total synthesis can tell us which enantiomer is the natural one.

### 1.2. Pyrrole-Imidazole Alkaloids Ecological Role

PIAs’ ecological role started to be investigated in the late ‘90s, when it was realized that sponges’ structural defences alone (spongin fibers and glass spicules) were ineffective feeding deterrents towards predatory reef fishes. The first report in this respect dates back to 1996, when an ecoassay-guided isolation, performed on the extracts of sponges of the genus *Agelas*, allowed the identification of 4,5-dibromopyrrole-2-carboxylic acid and oroidin (**1**) as the major components responsible for the observed chemical feeding deterrence [38]. Later on, stevensine, present in high concentration in the sponge *Axinella corrugata* (previously *Teichaxinella morchella*), was also demonstrated to possess antifeedant properties [39]. A structure-activity antifeedant relationship was subsequently established for oroidin-like molecules [40]. From these studies, the imidazole moiety, while not being active *per se*, looks like an enhancer of the deterrent activity exerted by the pyrrole counterpart. Other PIAs were recognized to chemically defend sponges from predators [41]. Acquarium and field feeding assays unveiled sceptrin (**8**) as the major metabolite of *Agelas conifera* able to deter fish feeding at natural concentrations, and it allowed ranking of a number of PIAs according to their antifeedant activities. This work, furthermore, highlighted the experimental observation that a higher number of bromine atoms present in the metabolite correlates with a higher feeding deterrent potency [42]. The ecological role of PIAs has been tentatively explained by a general interaction of those metabolites with the cellular calcium homeostasis [43,44]. From all those information, indicating that PIAs play a defensive role in sponges’ economy, still something is missing in our understanding. Combinations of PIAs did not provide any evidence of synergic activity [40]. Concentrations of some PIAs in the sponges are so low to have negligible deterrent effects [40,42]. Thus, the spontaneous question remains unanswered: “Why do sponges synthesize such a large number of alkaloids?”

Moreover, the isolation of both (+)-dibromophakellin (from *Pseudoaxynissa cantharella* [45]) and (−)-dibromophakellin (from *Phakellia flabellate* [25]) as well as (+)-dibromoisophakellin (from *Pseudoaxynissa cantharella* [45]) and (−)-dibromoisophakellin (from *Acanthella carteri* [46]) further complicates the scenario. As evolutionary pressure should select which enantiomer is better to perform a given function, do these enantiomeric alkaloids have a different ecological role in the biology of the sponge?

### 1.3. Pyrrole-Imidazole Alkaloids Classification

The 2-amino-4(5)-vinylimidazole portion of PIAs can vary with regard to its oxidative as well as hydration state, while the pyrrole-carboxamide moiety structural variations reside exclusively in the absence or the presence of bromine atom(s) in 2- and/or 3- position(s) of the pyrrole nucleus. The most commonly accepted PIA classification [2,9] is based upon both the number of “oroidin units” and the presence of extra-rings involving either the imidazole or the pyrrole portion of the alkaloids. Accordingly, five classes of PIAs will be encountered in the following sections:

1. Acyclic monomers
2. Cyclic monomers
3. Acyclic dimers
4. Cyclic dimers
5. Cyclic tetramers

Alkaloids whose structures hardly fit any of the aforementioned classification criteria have been included in a sixth additional class.

## 2. Acyclic Monomers

### 2.1. Oroidin

Oroidin (**1**) was isolated in 1971 from the marine sponge *Agelas oroides* [47]. It is the simplest among bromopyrrole-imidazole alkaloids and it has a linear structure characterized by a bromopyrrole carboxamide and an amino-imidazole moiety linked through a propenyl chain. Oroidin is the most abundant PIA and, as already mentioned (Subsection 1.1), it might be considered a biogenetic precursor of the other alkaloids.

Efforts towards the synthesis of oroidin followed two main strategies [2] employing either an already assembled imidazole ring [48–50] or an open chain precursor of imidazole [51,52]. In 2006, two total syntheses of oroidin were published, and both works have been recently reviewed [10].

**Scheme 1.**
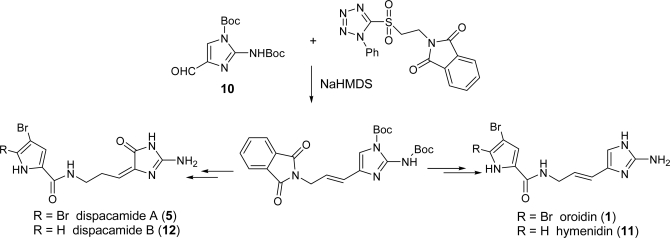
Approach to oroidin and related compounds *via* intermediate **10**.

Ando *et al.* [53] described a method involving a versatile intermediate **10**, arising from 2-aminoimidazol-4-carbaldehyde, that allowed the synthesis not only of oroidin (**1**) and hymenidin (**11**), but also dispacamide A (**5**) and dispacamide B (**12**). The general approach of the synthesis is reported in Scheme 1, while in Subsection 2.2 a full detailed version is described for the dispacamides.

**Scheme 2.**
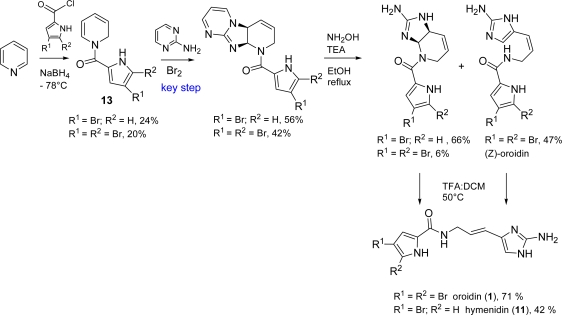
The Al Mourabit synthesis of oroidin.

Al Mourabit *et al.* [54], inspired by the natural product dibromoagelaspongine [19], performed a one-pot bromine-mediated oxidative addition of 2-aminopyrimidine, as a masked guanidine, on *N*-acyl-1,2-dihydropyridine **13**. The synthesis proved to be short and exquisite, with only little mass loss and good final yields (Scheme 2).

**Figure 3.**
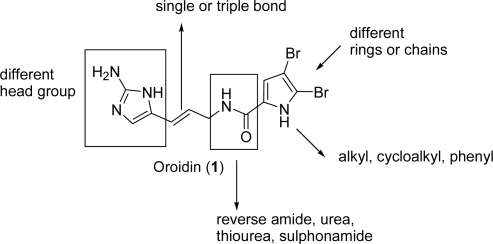
Examples of oroidin modification towards antibiofilm inhibitors.

The function of oroidin (**1**) as chemical defense for sponges of the genus *Agelas* against predation by the reef fish *Thalassomia bifasciatum* has already been mentioned (see subsection 1.2). Besides, **1** inhibits larval metamorphosis of the barnacle *Balanus amphitrite* (ED_50_ = 15 μg/mL) [21]. Oroidin was also reported to possess antibiofilm activity against the marine α-proteobacteria *R. salexigens* [55] and the medically relevant γ-proteobacterium *Pseudomonas aeruginosa* (PA; IC_50_ = 190 μM on PA01; IC_50_ = 166 μM on PA14) [56]. With the aim of finding new chemical entities able to inhibit the formation of bacterial biofilms [57–59], the simple and linear oroidin was proposed as a lead compound for SAR studies. Melander’s group synthesized several libraries of analogues based upon the oroidin template and the 2-aminoimidazole moiety (Figure 3).

Reverse amides [60,61], *N*-pyrrole substitutions [62], amide isosters [63] and linker modifications [64] are only few examples of a huge and successful synthetic work that led to the discovery of small molecules able to inhibit and disperse biofilms across order, class and phylum.

### 2.2. Dispacamide A

In dispacamide A (**5**), isolated in 1996 by Fattorusso *et al.* [65] from the sponge *Agelas dispar*, the oroidin 2-aminoimidazole moiety is oxidized to an alkylidene glycocyamidine. Dispacamide A is a potent, non competitive, antihistaminic agent, showing activity in the micromolar range on the guinea pig ileum [36,65] (pD_2_ 5.52 ± 0.11, where pD_2_ is the negative logarithm of the molar concentrations of the antagonists which induced a 50% decrease in the maximal response to the agonist). It also shows fish feeding deterrence (10 mM) [40].

Length and functionalization of the central linear chain are crucial parameters for the antihistaminic activity, whereas the presence of a bromine atom at position 2 of the pyrrole ring is not relevant. From a biogenetic point of view, dispacamide A is assumed [66] to be the direct precursor of pyrroloazepinone hymenialdisine (see Subsection 3.1).

**Scheme 3.**
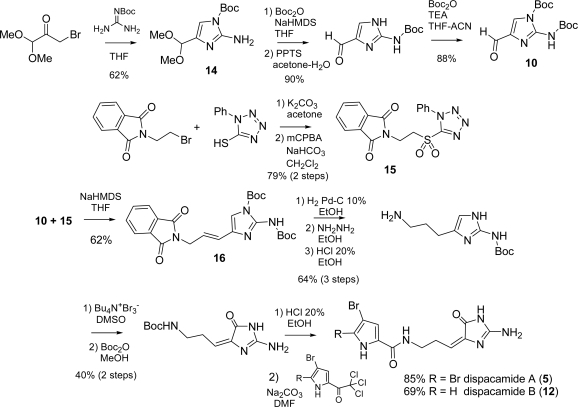
Ando’s synthesis of dispacamide A.

Several total syntheses of dispacamide A have hitherto been reported [51,66,67] and reviewed [4], in particular by Lindel *et al*. [2]. More recently, Ando *et al.* published a novel approach, which is described in Scheme 3 and was already mentioned in the oroidin subsection (see Scheme 1) [53]. In Ando’s strategy, the pyrrole part of the congeners for natural 2-aminoimidazole alkaloids was introduced at a later stage of the synthesis.

3-Bromo-1,1-dimethoxypropan-2-one and *tert*-butoxy-carbonylguanidine produced the functionalized aminoimidazole ring **14** in acceptable yield, which was then manipulated to furnish the suitably protected key intermediate 1,2-bis-*tert*-butoxycarbonylaminoimidazol-4-carbaldehyde (**10**).

The two building blocks **10** and **15** were then coupled *via* Julia olefination thus providing *E* olefin **16** in good yield and good selectivity. From **16**, the syntheses of oroidin (**1**) and hymenidin (**11**) were achieved (steps not shown), while hydrogenating the double bond paved the way for the preparation of dispacamides A (**5**) and B (**12**). The imidazole nucleus was oxidized with tetra-*n*-butylammonium tribromide and the primary amino group needed to be selectively Boc-protected for ease of purification. Final amidation with trichloroacetyl-pyrroles led to the desired dispacamides and could be used (in principle) to readily prepare derivatives with structural motifs differing from those involved in the natural products.

## 3. Cyclic Monomers

### 3.1. Hymenialdisine

**Figure 4.**
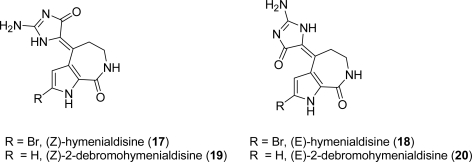
Structures of HMD and DBH.

(*Z*)-Hymenialdisine [(*Z*)-HMD; **17**, Figure 4] is the only PIA potently active as kinase inhibitor. As it showed nanomolar kinase inhibitory activity against a wide panel of kinases [68], it is potentially useful not only for the treatment of cancer, but also for illnesses such as Alzheimer’s disease and type 2 diabetes.

Nguyen and Tepe [69] recently published a review offering an exhaustive overview on several aspects of these molecules. (*Z*)-HMD (**17**) was originally isolated from marine sponges of the genera *Hymeniacidon, Acanthella, Axinella*, and *Pseudoaxinyssa*, while (*Z*)-2-debromohymenialdisine (DBH) (**19**) came from the sponge *Phakellia* [70].

(*Z*)-Hymenialdisines differ only by the presence of a bromine atom in the pyrrole α-position; these compounds share a fused bicyclic pyrrole[2,3-*c*]azepin-8-one linked through a double bond with a glycocyamidine ring. A few groups contributed to characterize HMD and DBH with spectral and X-ray studies [11,71,72].

Both the (*E*) and (*Z*) isomers have been isolated; they interconvert in a pH- and concentration-dependent manner [73] and (*Z*)-HMD (**17**) is the most abundant one, due to its higher thermodynamical stability.

**Scheme 4.**
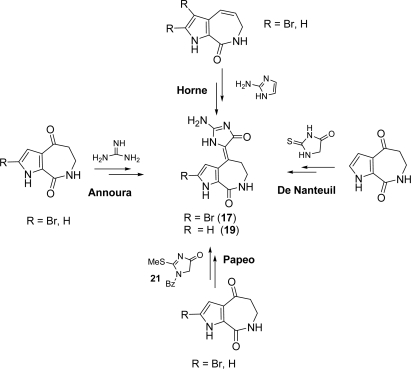
Synthetic approaches to (*Z*)-HMD (**17**) and (*Z*)-DBH (**19**).

Synthetic approaches [74–78] towards (*Z*)-HMD (**17**) and (*Z*)-DBH (**19**) have been reported (Scheme 4). 1-Benzoyl-2-methylsulfanyl-1,5-dihydroimidazol-4-one (**21**) employed in Papeo’s approach was also useful in the first total synthesis of (*Z*)-axinohydantoin (see Subsection 3.2).

Initially (*Z*)-HMD (**17**) was found to be slightly cytotoxic in PV4 cells (32.5% at 100 μg/ml) [79]. Meijer *et al*. extensively studied **17** from a structural and biological point of view [68]. (*Z*)-HMD was found to be a competitive inhibitor of ATP (Ki = 50 nM) and it proved to be a potent inhibitor of kinases like Cyclin-Dependent Kinases, Glycogen Synthase-3β and Casein Kinase 1 (Table 1).

The structure of a CDK2-HMD complex was determined at 2.1 Å resolution, indicating the binding of **17** in the ATP binding pocket [68]. The N1 atom of the pyrrole ring, the carbonyl oxygen and the amide nitrogen of the azepine ring form three hydrogen bonds with Glu81 and Leu83 of the CDK2 backbone. The pyrroloazepine bicyclic core is held in a shallow hydrophobic pocket by several van der Waals contacts with side chain atoms (31 of total 45). The bromine is partially exposed to the solvent and also packed against a few main-chain residues. In addition, the free NH_2_ group is involved in direct hydrogen bond formation with Asp145, while the glycocyamidine ring system makes other Van der Waals interactions together with two water-mediated hydrogen bonds with the main chain.

In spite of this high *in vitro* activity, **17** did not show significant inhibition in cells: only 40% of growth inhibition was reached at 100 μM in adenocarcinoma cells [1]. This behaviour might be ascribed to the low permeability of the molecule.

However, being potent *pan*-kinase inhibitors, HMD and analogues represent a potential for the treatment of neurodegenerative disorders, inflammatory pathologies, diabetes and cancer. Different series of HMD analogues were synthesized in order to establish a SAR [1,80,81]. In those derivatives, the main features that allow (*Z*)-HMD to bind in the kinase ATP binding site were left unchanged, while attempts have been performed in order to improve selectivity and permeability [82–84].

### 3.2. Axinohydantoins

**Figure 5.**
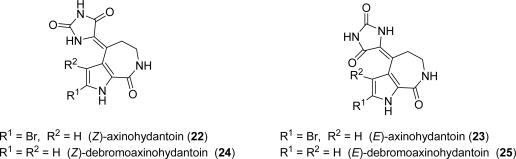
Axinohydantoins.

(*E*)-Axinohydantoin (**23**) and (*E*)-debromoaxinohydantoin (**25**) were isolated from the sponges *Axinella* sp. [85] and *Monanchora*, respectively (genera *Hymeniacidon*) (Figure 5) [86]. (Z)-Axinohydantoin (**22**) and (*Z*)-debromoaxinohydantoin (**24**) were subsequently isolated from the sponge *Stylotella aurantium* [87]. Those latter secondary metabolites were also found in *Hymeniacidon* species (Figure 5) [88].

Axinohydantoins are structurally related to HMD, but, in the former, the glycocyamidine ring is replaced by a hydantoin ring. While **22** displayed interesting micromolar inhibitory activity against a number of kinases (PKC, IC_50_ = 9 μM; GSK-3β, IC_50_ = 3 μM; CDK1/cyclin B, IC_50_ = 4 μM; CK1, IC_50_ = 4.5 μM; CDC5/p25, IC_50_ = 7 μM) [6,68,87], **24** inhibits, always in the micromolar range, only protein kinase C (IC_50_ = 22 μM). In addition, a slight activity was found on murine P388 lymphocytic leukemia for (*E*)-axinohydantoin (**23**) (ED_50_ of 18 μg/mL) [85].

The first total syntheses of **24** and **25** were reported by Horne *et al.* [89]. Recently our group accomplished the first synthesis of (*Z*)- and (*E*)-axinohydantoins (**22**) and (**23**), along with a second generation synthesis of **24** and **25** [90]. To this purpose, the chemistry already optimized for HMD synthesis [78] was successfully employed.

**Scheme 5.**
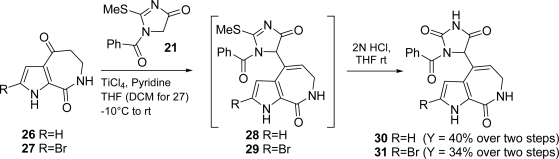
Northern ring installation.

Aldisine (**26**) and 2-bromoaldisine (**27**), available in multigram scale, were coupled with the versatile intermediate 1-benzoyl-2-methylsulfanyl-1,5-dihydroimidazol-4-one (**21**) to afford intermediates **28** and **29**, which were readily converted to the corresponding hydantoins **30** and **31** (Scheme 5).

**Scheme 6.**
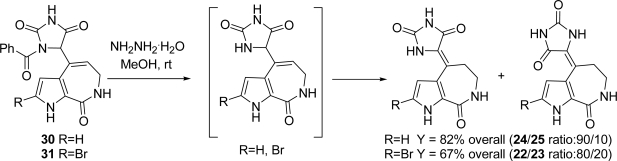
Benzoyl removal and double bond isomerization.

Intermediates **28** and **29** are also potentially suitable for preparing new analogues *via* nucleophilic displacement of the methylthio group. The cleavage of the benzoyl protecting group and the slow double bond *endo/exo* isomerization sequentially occurred in the presence of NH_2_NH_2_·H_2_O, thus delivering debromoaxinohydantoins (82% overall yield, **24**/**25** ratio: 90/10) and axinohydantoins (67% overall yield, **22**/**23** ratio: 80/20), respectively [90] (Scheme 6).

### 3.3. Dibromophakellstatin

**Scheme 7.**
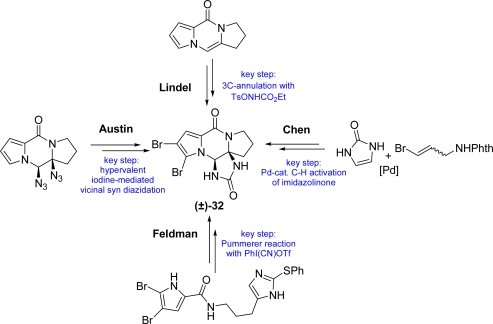
Racemic approaches to (±)-dibromophakellstatin **32**.

(−)-Dibromophakellstatin **32** is a tetracyclic monomeric pyrrole-imidazole alkaloid, isolated from the Indian Ocean sponge *Phakellia mauritiana* in 1997 by Pettit *et al.*, that showed interesting cell growth inhibitory activity against a minipanel of human cancer cell lines [91]. Since then, several total syntheses of the racemic natural product have been reported and reviewed [3,7,8,10]. The approaches by Lindel [92,93], Feldman [94,95], Austin [96] and Chen [97] for the construction of the tetracyclic core of *rac*-dibromophakellstatin are summarized in Scheme 7.

**Scheme 8.**
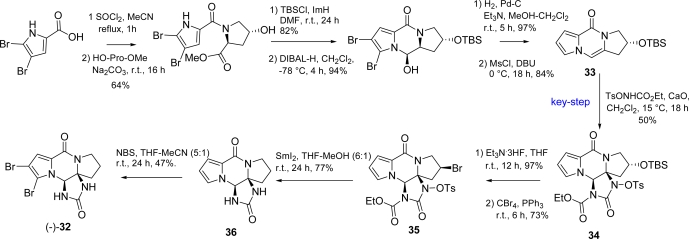
Enantioselective synthesis of (−)-**32**.

The first total synthesis of (−)-dibromophakellstatin **32** was accomplished by the Lindel group [98], who exploited the enantioselective version of their three-component imidazolinone annulation on a tricyclic enamide with TsONHCOOEt [92,93] (Scheme 8). Hydroxyproline was chosen as source of stereogenic information and used for the synthesis of chiral enamide **33**, where the TBS-protected hydroxy group, positioned in the preferred axial conformation, efficiently controlled the stereochemistry of the annulation, thus affording intermediate **34** as single diastereoisomer. The endgame to the natural product was put through by the reductive removal of hydroxy group after its conversion into alkylbromide **35** and treatment with SmI_2_ that, simultaneously, reduced the C-Br bond and deprotected imidazolone nitrogens to **36**. Final bromination with NBS afforded (−)-dibromophakellstatin **32**.

In late 2007, a careful investigation of the antitumor activity of dibromophakellstatin both as racemic mixture and as single enantiomers has been reported by Lindel [99]. *Rac*-dibromophakellstatin was tested on a panel of 36 human tumor cell lines and proved to be active on ovarian (IC_50_ = 0.60 μM), glioblastoma (0.93 μM), non-small cell lung (0.96 μM) and uterus (1.21 μM) cancer cell lines. When inhibitory activity against these cell lines was tested for single enantiomers, only (−)-**32** showed antitumor effects.

### 3.4. Agelastatins

**Figure 6.**
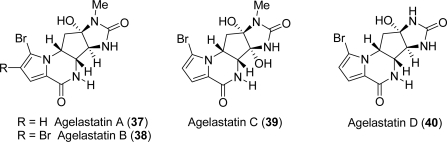
Agelastatins.

Four closely related compounds belong to this class of tetracyclic PIAs. Agelastatin A (**37**) and its brominated congener agelastatin B (**38**) were isolated in 1993 [20] from the marine sponge *Agelas dendromorpha* by Pietra and co-workers. They were fully characterized by using a combination of molecular modelling, NMR and exciton splitting [100]. Few years later two new metabolites, namely agelastatin C (**39**) and agelastatin D (**40**) were isolated from the extracts of the sponge *Cymbastela* sp. by Molinski and co-workers [101] (Figure 6).

These natural products display a densely functionalized tetracyclic core with four contiguous stereocenters, which is an enticing and synthetically challenging structural motif. Agelastatin A (**37**) is reported to exhibit significant biological activity [9]. After its recognition as cytotoxic agent towards KB cells at concentration below 1 μg/mL [20], Pietra and Pettit research groups showed a powerful activity of **37** against a wide range of cancer cell lines such as human KB nasopharyngeal cancer cells, L1210 murine tumor cell line, RT112/84 bladder carcinoma cells, SK-MEL-5 melanoma cells, HCT-116 colon carcinoma cells, and MDA-MB-435s breast cancer cells [37,68,102]. Comparative studies showed that agelastatin A inhibited tumor cell growth from 1.5 to 16 times more potently than cisplatin, particularly, against human bladder, skin, colon, and breast carcinomas [103].

Very recently, agelastatin A (**37**) was demonstrated to be very effective in down-regulating the expression of β-catenin and in up-regulating Tcf-4, an inhibitor of osteopontin (OPN) at the cellular level. These two effects result in repression of OPN and inhibition of OPN-mediated malignant cell invasion, adhesion, and colony formation *in vitro* [104]. Longley also highlighted that agelastatin A’s ability in inhibiting β-catenin, which also controls transcription from the multidrug resistance 1 gene [105], could be helpful in reducing drug resistance issues, possibly both as single agent as well as in combination therapy. Together with its cytotoxic activity, agelastatin A was also envisioned as inhibitor of glycogen synthase kinase-3 β (GSK-3β), an enzyme responsible for the neurofibrillary tangles typically found in Alzheimer’s disease, and as a mimetic of insulin [106].

The impressive biological activity, together with its scarce availability, prompted many synthetic chemists to engage in the total synthesis of **37**. To date, fourteen total syntheses have been achieved, each presenting different strategies to assemble the tetracyclic core of agelastatin A [11,107–111].

**Figure 7.**
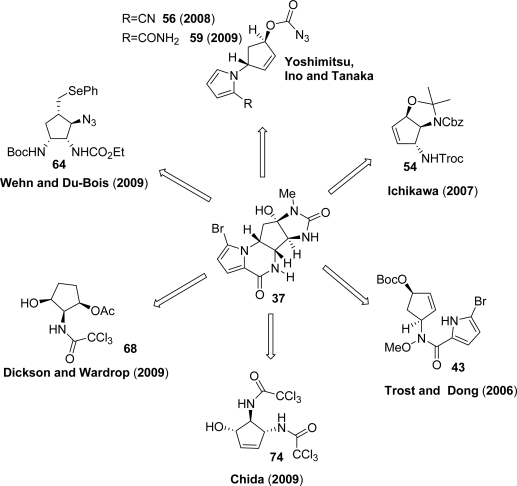
Different synthetic routes to achieve agelastatin A total synthesis.

In analogy to the previously published papers, the most recently reported syntheses of **37** show very different and elegant retrosynthetic analyses which represent a clear example of the creativity in the “art of total synthesis” [112]. The key structures involved in the achievement of agelastatin A synthesis are represented in Figure 7.

**Scheme 9.**
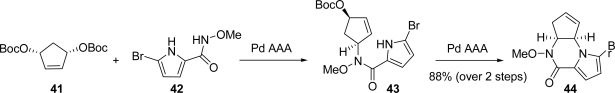
Tandem Pd-AAA reported by Trost and Dong.

Trost and Dong applied a metal-catalyzed asymmetric allylic alkylation (AAA) [113] to the synthesis of **37** by using pyrroles and *N*-alkoxyamides (hydroxamic esters) as nucleophiles [114,115]. Key intermediate **43** was obtained using an AAA between bisallylic carbonate **41** and the bifunctional nucleophile **42**. The challenging task, aimed at chemoselectively differentiating the two nucleophilic nitrogens in **42**, successfully allowed a tandem cyclization to obtain pyrrolopiperazinone **44** (Scheme 9).

**Scheme 10.**
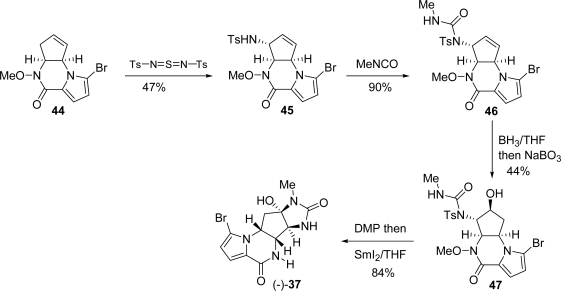
Completion of the Trost (−)-agelastatin A synthesis.

The Kresze reaction was used to carry out an allylic amination on **44**. Subsequent treatment of **45** with methyl isocyanate gave urea **46** (Scheme 10). Hydroboration followed by NaBO_3_-mediated peroxidation gave alcohol **47** which, after Dess-Martin periodinane (DMP) oxidation and protective group removal, afforded (−)-**37**.

**Scheme 11.**
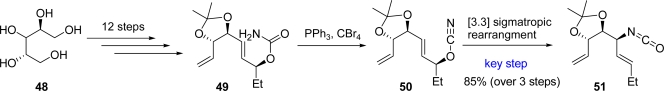
Allyl cyanate [3.3] sigmatropic rearrangment involving a [1,3] chirality transfer.

Ichikawa and co-workers achieved the total synthesis of (−)-**37** by using a strategy involving a [3.3] sigmatropic rearrangement of an allyl cyanate as the key step [116]. The synthesis started with an elegant transformation of L-arabitol **48** to obtain allyl carbamate **49**. Subsequent dehydration afforded allyl cyanate **50**, which rearranged to **51** with a [1,3] chirality transfer of the stereogenic center *via* a concerted six-membered transition state (Scheme 11).

**Scheme 12.**
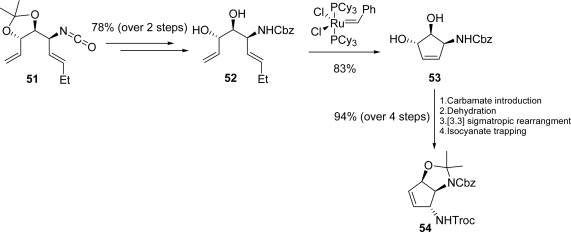
Access to key vicinal diamine **54**.

Further manipulations gave compound **52**, which was in turn subjected to ring-closing metathesis using first generation Grubb’s catalyst thus securing cyclopentene **53**. Introduction of the carbamate functionality followed by its dehydration delivered a substrate prone to a subsequent [3.3] sigmatropic rearrangement which, after isocyanate trapping, gave the vicinal diamine **54** (Scheme 12). The total synthesis of (−)-**37** was then completed in eight steps.

**Scheme 13.**
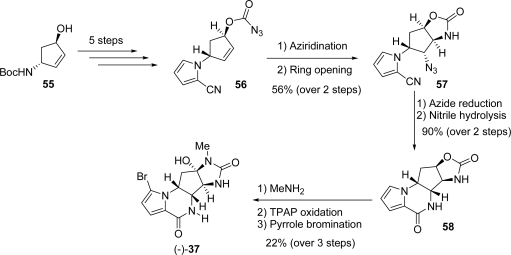
Aziridination reaction used in agelastatin A synthesis.

Yoshimitsu, Ino and Tanaka [117] reported the total synthesis of **37** starting from enantiomerically pure aminoalcohol **55** which was converted in the pivotal azidoformate **56** in five steps (Scheme 13). Vicinal dinitrogen functionalities were introduced through thermal aziridination of the π-bond followed by ring opening, thus obtaining azide **57**. Lactamization proceeded smoothly after azide reduction and nitrile hydrolysis affording carbamate **58**, which was opened using methylamine. The resulting alcohol was carefully oxidized using TPAP, and the derived ketone was trapped *in situ* by the vicinal urea nitrogen. This extremely concise synthesis was completed *via* pyrrole bromination (Scheme 13).

**Scheme 14.**
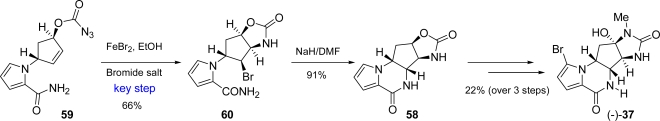
Yoshimitsu and Tanaka’s second-generation approach to (−)-**37.**

One year later the same research group proposed a second-generation approach affording a streamlined process to obtain agelastatin A (**37**) [118]. This synthesis is based on an iron halide-triggered radical aminohalogenation. Intermediate **59** was considered the most suitable substrate because of its restricted mobility, which might improve the stereochemical outcome of the reaction during the halogen transfer step. After extensive investigations, the authors reported that the best results were achieved by performing the reaction in ethanol with FeBr_2_ as radical initiator in the presence of bromide salts, such as LiBr or Bu_4_NBr. Azidoformate **59** was thus treated under the abovementioned conditions delivering bromide **60** in satisfactory yield, which was in turn reacted with NaH in DMF affording lactam **58**. Agelastatin A (**37**) was finally obtained in further three steps as outlined below (Scheme 14).

**Scheme 15.**
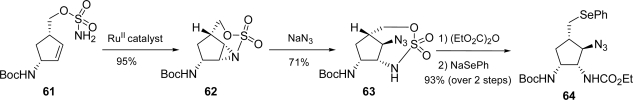
Synthesis of selenide key intermediate **64.**

Intramolecular olefin aziridination was used in 2009 by Wehn and Du Bois [119] to forge agelastatin A. In their synthesis, sulfamate **61**, easily prepared starting from commercially available materials, was subjected to aziridination in the presence of dimeric Ru^II^ catalyst in order to obtain **62**, which was then regioselectively opened to give oxathiazepane **63**. Interestingly, the catalyst was reported to be very effective, allowing very low loading (0.06 mol%) and high turnover number (>1500) thus enabling an easy and inexpensive scale-up. Key polyamine intermediate **64** was secured from **63** by treatment with ethylpyrocarbonate and then with NaSePh (Scheme 15).

**Scheme 16.**
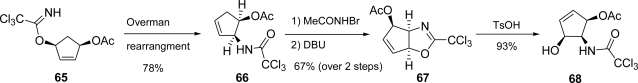
Synthesis of all *cis*-substituted cyclopentene **68.**

After construction of the central core, agelastatin A was obtained in seven steps. Racemic **37** was recently prepared by Dickson and Wardrop [120] using a synthetic route in which a trichloroacetamide group plays the simultaneous role of a protecting group, a pendant nucleophile assisting the cyclofunctionalization and the latent urea required for the imidazolinone ring construction. The key compound all *cis*-substituted cyclopentene **68** has been obtained starting from the imidate **65**, which after heating in xylenes, gave, through Overman rearrangement, trichloroacetamide **66**. Compound **67** was obtained upon treatment with *N*-bromoacetamide followed by DBU-mediated debromination. Exposure of dihydrooxazole **67** to *p*-toluenesulfonic acid gave key compound **68** (Scheme 16).

**Scheme 17.**
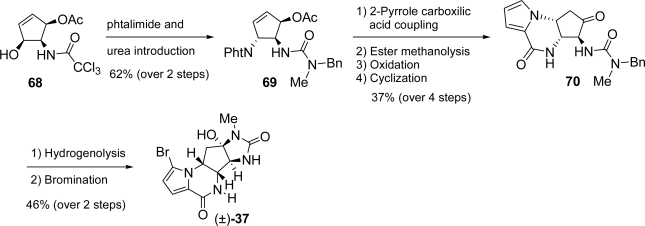
Completion of (±)-**37** synthesis according to Dickson and Wardrop.

Advanced intermediate **69** was easily obtained from **68** by converting the hydroxy group into the corresponding phthalimide under Mitsunobu conditions followed by installation of the urea moiety through displacement of the trichloroacetamide group. Tricycle **70** was forged by hydrazine-mediated phthalimide removal, coupling with 2-pyrrole carboxylic acid, acetate ester methanolysis, oxidation and base-mediated intramolecular cyclization. This synthesis was completed by removing the benzyl group with simultaneous imidazolidinone ring formation. Regioselective pyrrole bromination afforded (±)-**37** (Scheme 17).

**Scheme 18.**
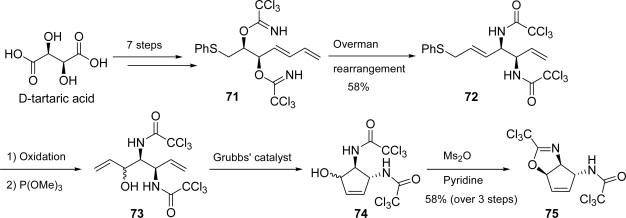
Synthesis of key compound **75.**

Chida and co-workers [121] envisioned diaminocyclopentene **74** as key intermediate for their agelastatin A synthesis. In order to obtain **74**, bis-trichloroimidate **71**, prepared in seven steps from commercially available d-tartaric acid, was subjected to a sequential Overman rearrangement. Allylic sulfide **72** thus obtained was oxidized to sulfoxide and, in the presence of P(OMe)_3_, underwent the Mislow-Evans rearrangement to diolefin **73**. Grubbs’ catalyst provided smooth access to **74**, which was finally converted into oxazoline **75** (Scheme 18).

**Scheme 19.**
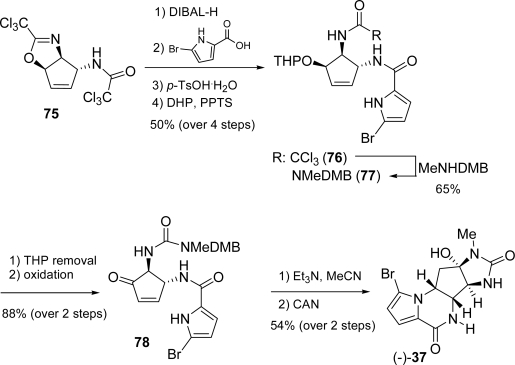
Chida and co-workers completion of (−)-**37** total synthesis.

Removal of the trichloroacetyl group in **75** with DIBAL, condensation of the resulting amine with 2-bromopyrrol-5-yl carboxylic acid, followed by hydrolysis of oxazoline and THP protection of the secondary alcohol afforded **76**. In order to carry out the aza-Michael addition to construct the piperazinone ring, intermediate **76** was converted into protected *N*-methylurea **77** by treatment with 2,4-dimethoxybenzylmethyl amine (MeNHDMB). Removal of THP and oxidation of the secondary alcohol gave α,β-unsaturated ketone **78** which, in turn, underwent the final aza-Michael addition. Targeted (−)-**37** was isolated in satisfactorily yield after oxidative cleavage of DMB group (Scheme 19).

## 4. Acyclic Dimers

### 4.1. Nagelamide D

**Scheme 20.**
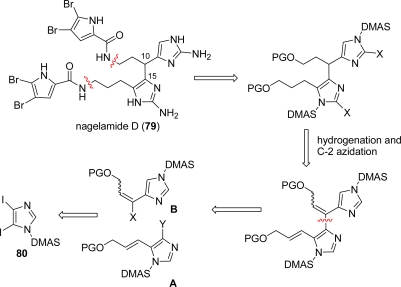
Retrosynthetic approach to Nagelamide D (**79**).

Nagelamide D (**79**) [122] has a connection between C-10 and C-15′ of its oroidin monomer units and was isolated as a racemate by Kobayashi’s group in 2003 from Okinawan marine sponges of the genus *Agelas.* Some antibacterial activity was reported against *Micrococcus luteus* (MIC, 4.17 μg/mL), *Bacillus subtilis* (MIC, 33.3 μg/mL) and *Escherichia coli* (MIC, 33.3 μg/mL). Nagelamide D (**79**) was synthesized by Lovely group [123] in 2009 according to the retrosynthetic pathway depicted in Scheme 20. The envisioned strategy foresees a cross-coupling reaction between imidazolyl fragment **A** and vinyl fragment **B**, both accessible from a common precursor, the protected diiodoimidazole **80** [124].

**Scheme 21.**
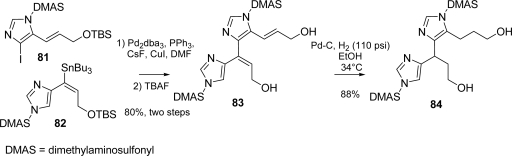
Nagelamide D: Fragment Assembly and Reduction.

The two actual fragments **81** and **82** were coupled according to the Baldwin procedure [125] for fluoride-mediated Stille reaction, providing bis-vinylimidazole **83** in good yield after treatment with TBAF to complete the partial desilylation. The next step was a catalytic hydrogenation to saturate both double bonds (Scheme 21).

**Scheme 22.**
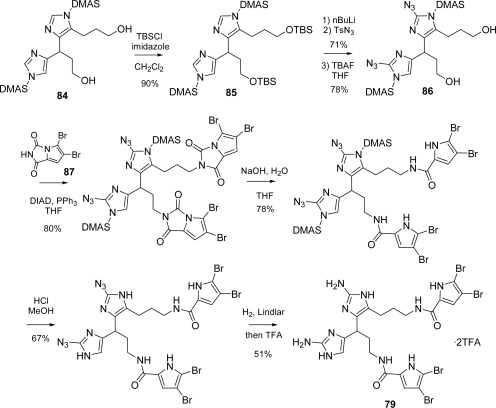
Completion of the synthesis of nagelamide D (**79**).

Diol **84** was then protected as bis *t*butyl-dimethylsilylether **85** and then transformed into the bis azide **86**, before desilylation of the hydroxy groups with TBAF (Scheme 22). At this point, the two bromopyrrole moieties were introduced by means of a double Mitsunobu reaction with dibromopyrrolohydantoin derivative **87**, which completed the formation of the nagelamide D full skeleton. The pyrrolohydantoin rings were then hydrolyzed, followed by imidazole deprotection with methanolic HCl. Azides were finally reduced over Lindlar catalyst affording nagelamide D (**79**), which was isolated as its TFA salt.

The authors reported that ^1^H-NMR of synthetic nagelamide D did not match that of the naturally occurring material; moreover, they were unable to get original NMR data from Kobayashi in order to unravel this inconsistency. Lovely also mentioned [123] an unpublished biomimetic total synthesis of nagelamide D by Horne *et al.*, whose spectroscopic data perfectly matched the ones from Lovely group. The question whether the assigned structure or Kobayashi’s NMR data are in error remains open (on the subject see also Usami [126]).

### 4.2. Sceptrin

**Figure 8.**
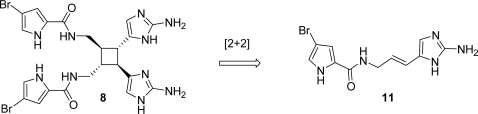
Proposal for the biogenetic origin of sceptrin (**8**).

Sceptrin (**8**) (Figure 8) was isolated in 1981 by Faulkner and Clardy [127] from *Agelas sceptrum.* It is a dimeric pyrrole imidazole alkaloid formally made up by two hymenidin (**11**) subunits. The biogenetic hypothesis derives sceptrin from hymenidin (**11**) *via* an enzyme-mediated [2+2]-cycloaddition [127].

**Scheme 23.**
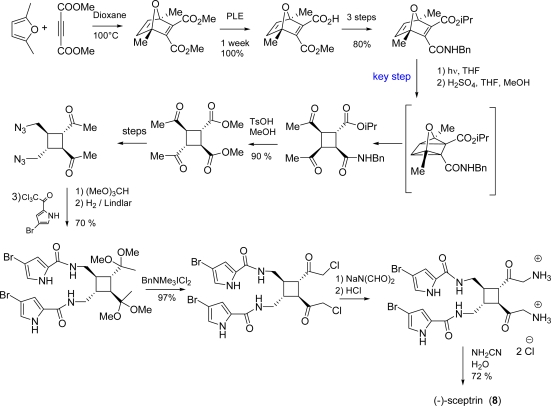
Baran’s total synthesis of (−)-sceptrin (**8**). PLE = pig liver esterase, DMT-MM = 4-(4,6-dimethoxy[1,3,5]triazin-2-yl)-4-methylmorpholinium chloride.

After some unfruitful attempts to synthesize sceptrin from urocanic acid derivatives [128], Baran [129] and Birman [130] independently reported the synthesis of racemic sceptrin in 2004; two years later, the first enantioselective synthesis of (−)-sceptrin was published (Scheme 23) [131]. Main hallmarks [132] of this approach are:

- minimal use of protecting groups (benzylamide proved to be crucial for a complete transfer of chirality);
- application of an oxaquadricyclane rearrangement/fragmentation [133] in natural product synthesis to diastereoselectively access the cyclobutane core of sceptrin;
- application of a new chemo- and regioselective halogenation method [134];
- formation of 2-aminoimidazole in mild conditions [135] (these moieties were voluntarily introduced at a later stage of the synthesis because the earlier introduction of this step resulted in compounds intractability).

Sceptrin has useful biological properties, like antibacterial and antiviral [136], antihistaminic and antimuscarinic activity [137–139]; besides, it is a natural inhibitor of somatostatin [140]. Recently, Rodriguez and his group, through bidirectional affinity experiments, identified sceptrin as being able to bind to MreB [141], a cell wall regulator, and thus a validated antibiotic target [142]. When sceptrin interacts with MreB, cell wall disruption was observed.

## 5. Cyclic Dimers

### 5.1. Ageliferin

**Figure 9.**
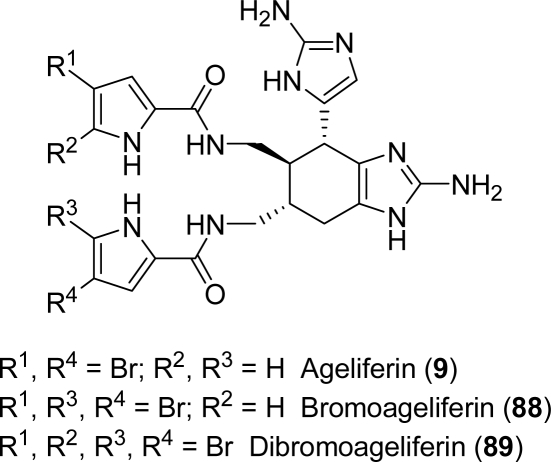
Ageliferins.

Ageliferins (**9**, **88** and **89**, Figure 9) are dimeric pyrrole-imidazole alkaloids that have been isolated from the sponge *Agelas conifera* [136, 143]. This family of natural products showed antimicrobial and antiviral activity [136] as well as actomyosin ATPase activation [144]. Ageliferins, like the majority of alkaloids originated by sponges of the genus *Agelas*, are feeding deterrents.

**Figure 10.**
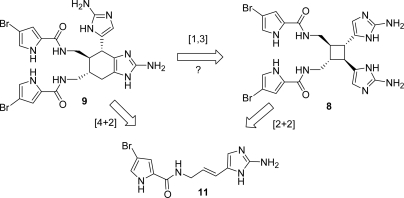
Proposal for biogenetic origin for ageliferin (**9**).

The chemical defence that sponges adopt against predatory reef fishes has been investigated and measured [42], revealing that ageliferins, together with their close relatives sceptrins, are responsible for deterrence in the sponge *Agelas conifera*. Biogenetically, ageliferin (**9**) was hypothesized to derive from hymenidin (**11**) *via* an enzyme-mediated [4+2]-cycloaddition that infers chirality to achiral precursors, as well as an enzyme-catalyzed [2+2]-cycloaddition generates sceptrin (**8**) [29] (Figure 10).

**Figure 11.**
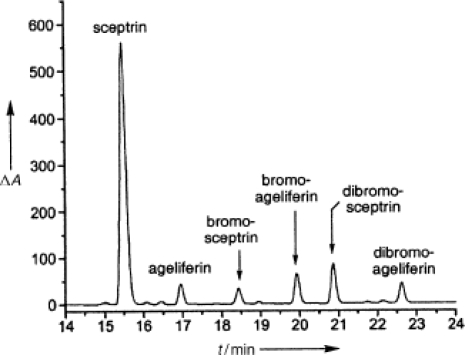
Extracts composition of *Agelas conifera*. Reproduced with permission from Verlag *Z. Naturforsch.* [145].

Since its isolation and identification, several synthetic efforts aiming at the synthesis of **9** were based on this biogenetic hypothesis [3]. In 2004 Baran and co-workers speculated about the extracts composition of *Agelas conifera* [145] (Figure 11), in which sceptrin was dramatically more abundant than ageliferins [26]. If the two families of alkaloids derived from the same linear precursor *via* two different cycloaddition pathways, ageliferins should be thermodynamically more abundant than sceptrins in the extract.

**Scheme 24.**
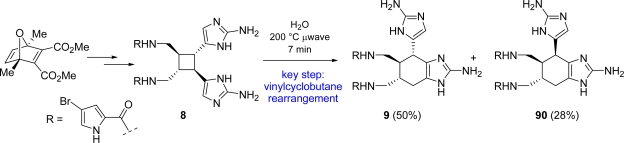
Enantioselective synthesis of (−)-ageliferin (**9**) and (−)-nagelamide E (**90**).

An alternative biogenetic pathway was then postulated involving a formal [1,3]-sigmatropic rearrangement of sceptrin followed by a double bond isomerisation [26, 132] (Figure 10). According to this hypothesis, the vinylcyclobutane of *rac*-sceptrin was efficiently converted into the cyclohexene core of *rac*-ageliferin under microwave irradiation and some speculations on the reaction mechanism have been reported [26]. Two years later, Baran and co-workers published the accomplishment of the total synthesis of (−)-sceptrin (**8**), whose subsequent microwave-mediated vinylcyclobutane rearrangement yielded the naturally occurring (−)-ageliferin (**9**) (40% yield along with unreacted sceptrin) [131]. The scale-up of the reaction and the prolonged heating resulted in the formation of *epi*-ageliferin, later christened nagelamide E (**90**) [122], which was synthesized for the first time [132, 146] (Scheme 24).

Among the mechanistic speculations originally reported [26], the hypothesis of a 6-*endo*-*trig* recombination and olefin isomerization of the diradical intermediate deriving from the radical scission of the cyclobutane ring, showed to be more consistent with the observed partial stereochemical erosion.

**Scheme 25.**
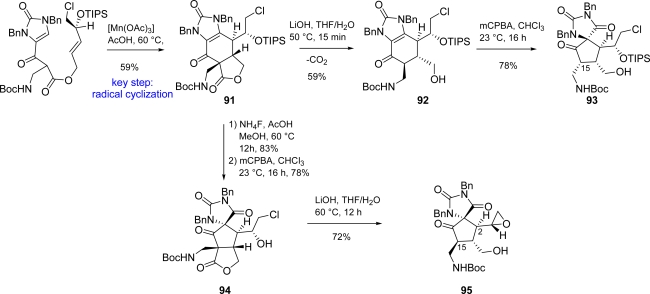
Chen approach to oroidin cyclic dimers core.

A different approach to the core skeleton of ageliferin, as well as other oroidin cyclic dimers, has been reported by Chen *et al*. in 2006, who exploited a regiocontrolled Mn^III^-mediated oxidative heterobicyclization of a β-ketoester for the construction of tricycle **91**. The reaction proceeded through a *5-exo/6-endo* radical cyclization pathway [147] (Scheme 25).

Hydrolysis of **91** revealed ageliferin core **92**, which was oxidatively rearranged to massadine skeleton **93** despite epimerization at C15. Lactone **91** can be oxidized after removal of the TIPS protective group, generating the spiro-derivative **94** with opposite facial selectivity. Subsequent hydrolysis afforded intermediate **95**, which bears the palau’amine spiro-skeleton, but opposite relative configurations at C2 and C15.

**Scheme 26.**
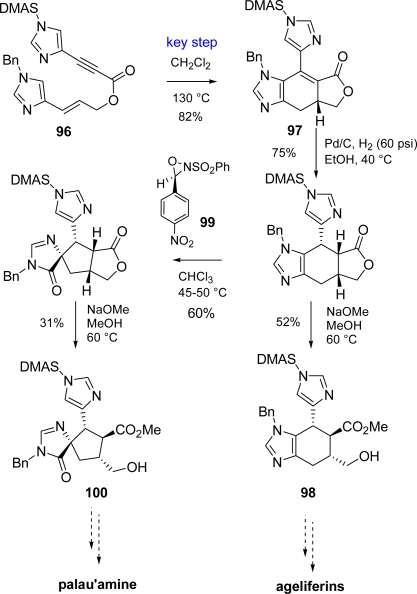
IMDA approach to ageliferins and palau’amine cores.

In mid-2009 an alternative approach has been described by Lovely [148], who exploited a thermal intramolecular Diels-Alder (IMDA) on enyne **96** for the construction of the intermediate **97**, which could be converted to ageliferins core **98** or, in turn, rearranged under oxidative conditions in the presence of Davis’ oxaziridine (**99**) to the palau'amine nucleus **100** (Scheme 26).

### 5.2. Palau’amine

**Figure 12.**
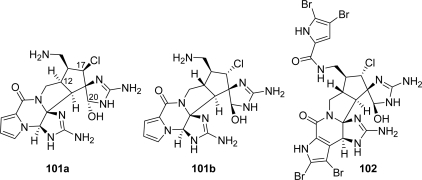
Previously assigned (**101a**) and revised (**101b**) structure of palau’amine.

The hexacyclic bisguanidine palau'amine (**101)** was isolated in 1993 by Scheuer and co-workers [149] from the sponge *Stylotella agminata*. It showed cytotoxicity against some tumor cell lines as well as antibiotic and antifungal activity. The relative configuration initially assigned (**101a**) to the structure implies a *cis* junction of the azabicylo[3.3.0]octane moiety and a *cis* relationship between the chlorine atom and the aminomethyl chain (Figure 12).

Later, while reporting the isolation and structure elucidation of tetrabromostyloguanidine (**102**) (also named carteramine A), Matsunaga [150] and Köck [151] raised the question for a revision of the assigned structure of palau'amine, according to NMR data and computational experiments on related compounds. Finally, in 2007 Quinn *et al.* [152] reported the correct structure of palau'amine (**101b**), entailing an inversion of configuration at C12, C17 and C20 (Figure 12) resulting in a thermodynamically less obvious *trans-*fused azabicylo[3.3.0]octane moiety and with the chlorine atom being *trans* to the vicinal aminomethyl chain.

**Scheme 27.**
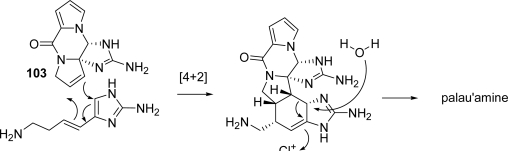
Scheuer's proposal for biogenesis of palau'amine.

In 1998 Scheuer [22] proposed palau'amine as biogenetically arising from a Diels-Alder reaction between 11,12-dehydrophakellin (**103)** and 3-amino-1-(2-aminoimidazolyl)prop-1-ene, followed by a chloroperoxidase-catalyzed chlorination that initiates a 1,2-shift/ring contraction and water addition (Scheme 27).

**Figure 13.**
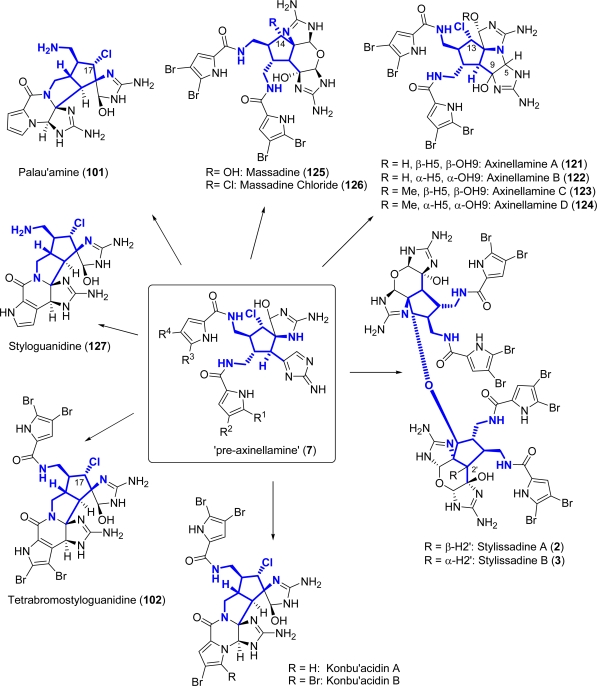
Structurally related PIAs cyclic dimers.

The revision of the structure of **101** exerted a profound influence on the biogenetic hypothesis concerning dimeric PIAs (see Subsection 1.1 and Figure 13).

Since its isolation, efforts towards the total synthesis of palau'amine have been increasing [3]. Recently Baran *et al*. have reported the total synthesis of axinellamines [153] and massadines [154]. These achievements may represent the prelude for the completion of their own strategy toward palau'amine.

Lovely exploited an intramolecular Diels-Alder approach (IMDA) for the construction of the intermediate **97** (see Scheme 26) that, after stereoselective double bond reduction, was subjected to an oxidative rearrangement thus securing the spiro-fused system of palau'amine (**100)**. To this purpose Davis' reagent (**99**) [148] delivered better results than its ancestor dimethyldioxirane [155].

Chen reported a regiocontrolled Mn^III^-mediated oxidative heterobicyclization of a β-ketoester yielding the spiro intermediate **95** after suitable modifications [147] (Scheme 25).

Romo succeeded in the synthesis of the *trans-*fused azabicyclo[3.3.0]octane core of palau'amine by exploiting a biomimetic chlorination and a concomitant 1,2 shift/ring contraction of **104** for the construction of *cis*-fused intermediate **105** [27,156,157].

**Scheme 28.**
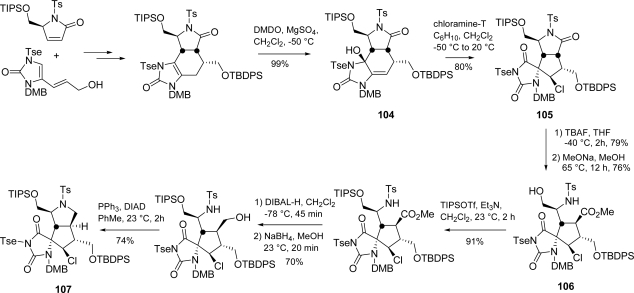
Romo’s approach to palau’amine core **107**.

Subsequent selective deprotection, followed by simultaneous ring cleavage/epimerization with sodium methoxide afforded the *anti*-substituted cyclopentyl ester **106**. Further manipulations yielded *trans*-azabicyclo[3.3.0]octane **107** [158] (Scheme 28).

The tricyclic prolinol derivative **107** was envisioned to be a suitable substrate for the application of the oxidative annulation strategy that recently provided the first enantioselective synthesis of (+)-phakellin from prolinol by Romo’s group [159]. This approach would lead to the targeted palau'amine (**101**).

Overman’s approach foresees thiosemicarbazide annulation on a densely functionalized 4,5-dihydropyrrole-2-carboxylate **108** as the key step for the assembly of a triazatriquinane **109**, precursor of the *cis*-fused tetracyclic skeleton of *epi*-palau'amine [160]. Evolution of intermediate **109** by means of a SmI_2_-mediated N-N bond reduction yielded **110**.

**Scheme 29.**
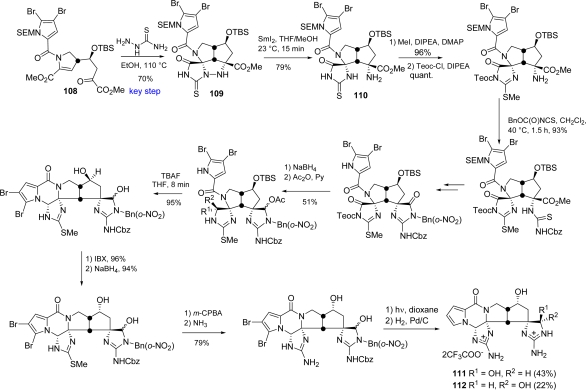
Overman’s synthesis of congeners **111** and **112** of *epi*-palau’amine.

The following insertion of a spiro glycocyamidine ring, a subsequent TBAF-promoted cyclization for the assembly of the ketopiperazine moiety and further manipulations, resulted in the synthesis of hexacyclic congeners **111** and **112** of *epi*-palau’amine, bearing a *cis* configuration at the azabicyclo[3.3.0]octane core [161] (Scheme 29).

Harran exploited a different approach to palau'amine following his biogenetic proposal which was based upon a spirocycloisomerization of tethered alkylidene glycocyamidines [23]. For this purpose, tetrahydropyridazine (**113**) has been acylated with acyl chloride **114** [160] and subsequently elaborated to intermediate **115**, from which target monomer **116** was derived by mild thermolysis in the presence of HgCl_2_.

**Scheme 30.**
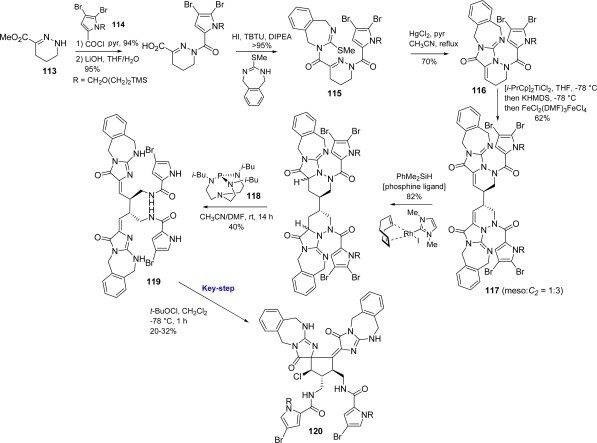
Harran's approach to palau'amine spirocyclic core **120**.

Treatment of **116** with (*i*-PrCp)_2_TiCl_2_ prior to KHMDS, followed by exposure to FeCl_2_(DMF)_3_FeCl_4_, generated **117** as mixture of meso and *C_2_* isomers. Hydrosilylation of **117**, performed on the *C_2_* isomer, and reduction mediated by proazaphosphatrane **118** yielded the partially debrominated bis-alkylidene derivative **119** that underwent spirocyclization in the presence of *t*-BuOCl affording the spirocyclic core of palau'amine **120** [162] (Scheme 30).

### 5.3. Axinellamines

Like palau’amine (**101**), axinellamines (**121**–**124**), massadines (**125**, **126**) and stylissadines (**2**, **3**) (Figure 13) are PIAs with a high degree of complexity and for this reason they are appealing targets for total synthesis.

Axinellamines (**121**–**124**) were first isolated from *Axinella* sp. [163]. Their architecture is characterized by important stereogenicity (8 contiguous stereocenters) and a fully decorated cyclopentane ring. Moreover, axinellamines B-D (**122**–**124**) display antibacterial activity against *Helicobacter pylori*, a bacterium implicated in pepticular and gastric cancer (minimum inhibitory concentration (MIC) for bactericidal action against *H. pylori* at 1000 *μ*M) [163]. Several papers have been published reporting synthetic efforts toward the synthesis of axinellamines and some of them have already been reviewed [2,3,7,147,164–166].

**Figure 14.**
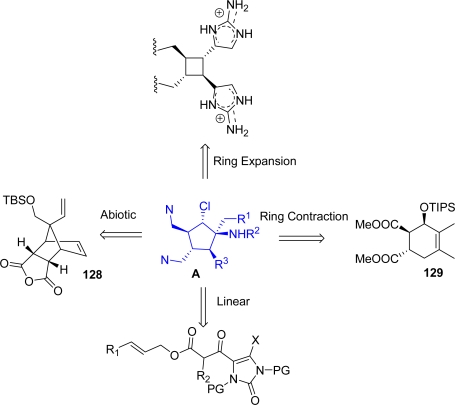
Different approaches toward intermediate **A**.

The key intermediate in the synthesis of axinellamines is the elaborated cyclopentane ring **A** (Figure 14), also common to palau’amine, massadine, styloguanidine (**127**, Figure 13) [167] and stylissadines. The first enantioselective synthesis toward an analogue of **A**, which exploited the desymmetrization of **128**, was reported by Carreira *et al*. in 2000 [165].

Many approaches to this intermediate were envisioned and they can be classified into four main categories [30] (Figure 14): a) linear [23,147], b) ring contraction, [27,129,132,146,156,157,166, 168–171], c) ring expansion (this approach still awaits experimental realization) [132] d) abiotic [160,165, 172–174].

**Scheme 31.**
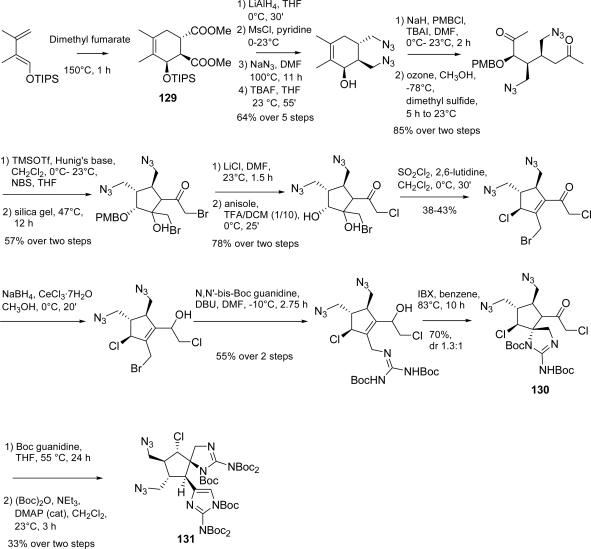
Baran synthesis of key intermediate **131**.

**Scheme 32.**
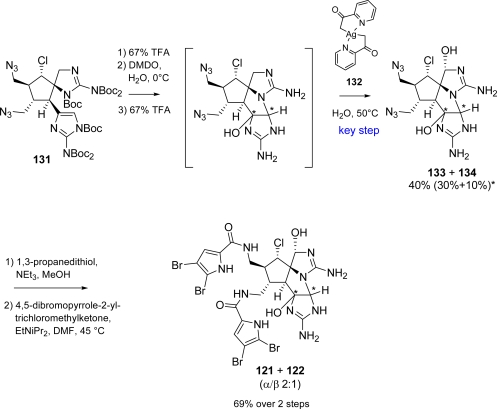
Completion of the axinellamine A (**121**) and B (**122**) total synthesis. * 77% (H, OH=β) and 48% (H, OH= α) after optimization: **132**, H_2_O, TFA 10% (v:v), r.t^155^

The first total synthesis of axinellamine A (**121**) and B (**122**) was completed by Baran *et al*. [153] in 2008 starting from **129** and using an extremely straightforward approach. Key step was an oxidation (by means of Ag^II^ complex **132**, Scheme 32), on polyfunctionalized intermediate **131**. Both regioselectivity and overoxidation control of this reaction proved to be very impressive [34,153]. Synthesis of key intermediate **131** required 17 steps with an overall yield of 1.3% (Scheme 31).

Starting from advanced intermediate **131**, the synthesis of **121** and **122**, as racemic mixture, was then completed in 6 steps and an overall yield of approximately 0.3% (Scheme 32).

### 5.4. Massadines

Starting from **130**, Baran *et al*. synthesized also massadine (**125**) and massadine chloride (**126**), a precursor of the daunting stylissadines (Figure 13).

Massadine was first isolated in 2003 from the marine sponge *Stylissa* aff. *Massa* [34]. Like the axinellamines, it is a dimeric pyrrole-imidazole alkaloid. Its peculiarity is due to the presence of a hydroxy group at C14 that renders this compound unique among the dimeric PIAs (Figure 13). Massadine (**125**) is biologically active as inhibitor of geranylgeranyltransferase type 1 from *Candida albicans* with IC_50_ of 3.9 μM [34]. A postulated biosynthetic precursor of **125** is massadine chloride (**126**), first isolated from *Stylissa caribica* [28]. Massadine chloride possesses a chlorine atom at C14, as in axinellamines (**121**–**124**) (C13), palau’amine (**101**) (C17) and tetrabromostyloguanidine (**102**) (C17) [151] (Figure 13).

**Scheme 33.**
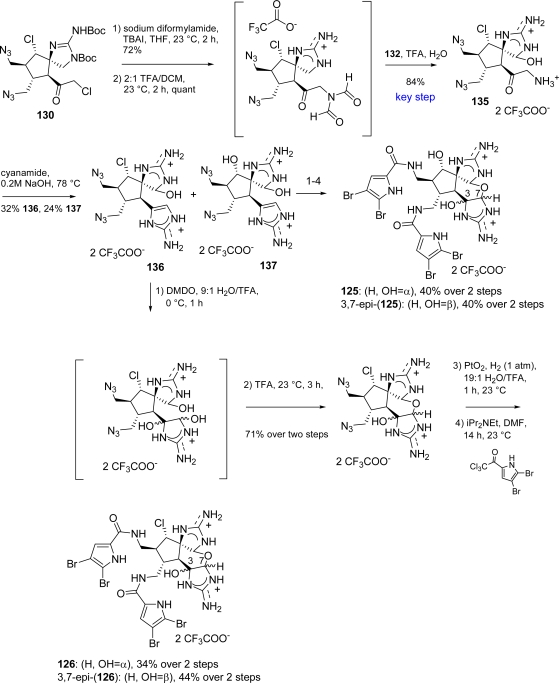
Synthesis of **125** and **126**.

Syntheses of these alkaloids proved soon to be challenging due to the presence of many functional groups leading to several failed approaches [154]. Finally, **125** and **126**, together with their unnatural C3,C7 epimers succumbed to total synthesis as reported in Scheme 33[154].

The key step of the synthesis was again the silver(II)-mediated oxidation reaction already reported in the synthesis of axinellamines (see Scheme 32). An optimization of the reaction conditions was necessary for massadines synthesis and led to the finding that TFA accelerates the oxidation, thus providing a general method to chemoselectively oxidize unprotected guanidines. Also the axinellamines synthesis overall yield was improved, obtaining **133** and **134** (Scheme 32), in shorter reaction times and at room temperature. The subsequent treatment of **135** with cyanamide required controlled pH conditions giving **136** along with its hydroxy analogue **137** (Scheme 33). The same reaction sequence was performed on both **136** and **137** leading to massadine chloride (**126**) and massadine (**125**) respectively, although each one associated with the corresponding C3,C7-epimer. Both **126** and 3,7-*epi*-(**126**) can be converted to their hydroxy analogues, **125** and 3,7-*epi*-(**125**), in warm water, probably involving a nucleophilic substitution mechanism with retention of configuration *via* a massadine aziridine species [28].

## 6. Cyclic Tetramers

### Stylissadines

Stylissadines (**2** and **3**, Figure 1 and 13) are tetrameric members of PIAs. Structurally, they are ether-linked dimers of massadine and thus the largest and the most complex structures within the oroidin family of alkaloids discovered so far. Stylissadine A (**2**) is the formal condensation product of two molecules of massadine (**125**) and it has a *C*_2_ symmetry. Stylissadine B (**3**), on the contrary, does not have a *C_2_* symmetry because the C-2’ stereocenter is inverted [175]. Both have biological activity (**2**: IC_50_ = 0.7 μM; **3**: IC_50_ = 1.8 μM) as antagonists of the P2X7 receptor involved in inflammatory diseases, but their high molecular weight and structural complexity render them hard to develop as drugs [176]. Efforts to convert massadine chloride into stylissadines are underway in Baran’s group.

## 7. Others

### Ageladine A

In 2003, Fusetani *et al.* isolated the fluorescent alkaloid ageladine A (**138**) from the sponge *Agelas Nakamurai* by a bioassay-guided extraction [177]. Ageladine A is reported to inhibit several matrix metalloproteinases (MMPs-1, -2, -8, -9, -12 and -13). As it has been demonstrated that **138** does not chelate zinc ion like all known MMP-inhibitors do, the natural product probably operates with a completely different mechanism of action. Ageladine A showed also antiangiogenic effects. Moreover it is the first isolated PIA containing a 2-aminoimidazolopyridine moiety.

**Figure 15.**
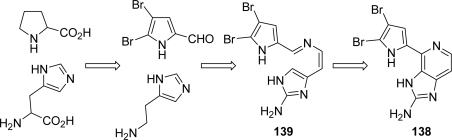
Proposed biogenesis for ageladine A (**138**).

In the same paper, Fusetani also hypothesized a possible biogenesis of ageladine A: amino acids proline and histidine evolve to dibromopyrrole carboxyaldehyde and histamine, respectively. They subsequently generate intermediate imine **139**, which undergoes oxidative intramolecular cyclization to afford **138** (Figure 15).

**Figure 16.**
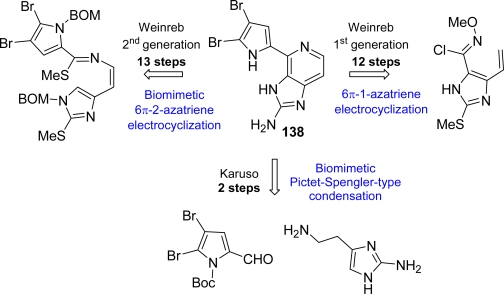
Approaches to ageladine A (**138**).

Since its isolation, quite a few total syntheses have been reported and recently reviewed [7,8,10]. As shown in Figure 16, the first generation Weinreb approach toward **138** settled a 6π-1-azatriene electrocyclization as the key step [178,179], while a biomimetic synthesis was reported one year later by the same group exploiting a 6π-2-azatriene electrocyclization [180,181]. In the meantime Karuso *et al.* published an elegant, two-step, biomimetic synthesis of ageladine A starting from 2-aminohistamine and 4,5-dibromo-2-formylpyrrole and moving through a Pictet-Spengler-type condensation [182].

An evaluation of the MMP-12 inhibition efficiency of ageladine A and its analogues has been reported by Ando and co-workers [183], confirming that the two bromine atoms and the NH groups are essential for the biological activity. Moreover very recently it has been demonstrated that **138** is a reliable and stable fluorescent pH sensor. Because of its permeability it can be used for detection of intracellular pH changes [184].

## 8. Conclusions

Pyrrole-imidazole alkaloids (PIAs), a quite enlarged family of metabolites exclusively found in marine sponges, fascinate the scientific community for several reasons. Although the isolation of the first representatives of this family dates back to the ‘70s, new relatives continue to be unveiled, thus requiring a parallel fine-tuning in the speculations around their biogenesis and their ecological role in the sponges. Moreover, the quest to support hypotheses with experimental data, by increasing our knowledge in sponges’ biochemistry, proved to be encumbered by the difficulties in artificial sponge cell culturing. Challenges are also plentiful from a synthetic point of view. Architectural complexity, unusually high nitrogen content (N/C ≅ 1:2), structural revisions, unknown absolute stereochemistry are just some of the dreadful aspects one has to take into account while planning a total synthesis of these alkaloids. Those synthetic efforts however should not be considered just as simple intellectual *divertissements*: some PIAs are gifted with promising biological activities, and larger availability of these compounds is key in developing more accurate pharmacological profiles.

## Figures and Tables

**Table 1.** Kinase inhibition selectivity of 17.

**Enzyme**	**IC_50_ (nM)**	**Enzyme**	**IC_50_ (nM)**

CDK1/cyclin B	22	Erk 1	470
CDK2/cyclin A	70	Erk 2	2000
CDK2/cyclin E	40	c-raf	>10,000
CDK3/cyclin E	100	MAPKK	1200
CDK4/cyclin D1	600	GSK-3β	10
CDK5/p25	28	CK1	35
CDK6/cyclin D2	700	CK2	7000
